# Personal

**Published:** 1906-11

**Authors:** 


### PERSONAL.

Dr. John McGaw Woodbury, late Major and Chief Surgeon United States Volunteers, who since the war with Spain, or for a good portion of the time, has been Commissioner of Street Cleaning in the city of New York, has resigned that office because he would not permit his department to be debauched by politics.

Dr. Woodbury is easily the best head of the street cleaning department New York has ever employed,—a statement that may be made without disparagement to any of his predecessors,—and it is to be regretted that he must be sacrificed to the clamor of the political machine.

---

Dr. W. E. Fitch, editor of *Gaillard's Southern Medicine,* has removed from Savannah, Ga., to New York, his address being at 21 West 97th Street, where he will continue his editorial and professional work.

---

Dr. Roswell Park, of Buffalo, has been elected to fellowship in the Association of French Surgeons. He is the fourth member, according to the *Buffalo Express,* to be elected to that society in the United States.

---

Brigadier General Robert M. O'Reilly, M.D., Surgeon General of the United States Army for the past four years, was reappointed to that office September 7, 1906, that being the date of the expiration of his first term of service as the head of the medical department of the army. General O'Reilly will reach the age for retirement in 1909, hence will have more than two years to serve in his present capacity.

---

Dr. H. R. Gaylord, of Buffalo, fractured his leg recently in an automobile accident, for which he is under treatment in a hospital at Detroit.

---

Dr. Wisner R. Townsend, of New York, Secretary of the Medical Society of the State of New York, spent the day in Buffalo, October 8, 1906, and was present in the evening at the meeting of the Medical Society of the County of Erie. He made some felicitous remarks relating to the work of amalgamation of the two state organisations and their constituent county societies. Erie county was the last to complete the affiliation.

---

Major Paul F. Straub, formerly surgeon of the 36th U. S. Volunteer Infantry, while on duty with the regiment December 21, 1899, at Alos-Zambales, Island of Luzon, P. I., carried a wounded soldier to a place of safety in the face of the enemy's fire. For this act of bravery he was commended at the time by General J. Franklin Bell and on October 6, 1906, President Roosevelt conferred upon Major Straub a medal of honor. The ceremony took place at the White House, in the presence of a distinguished gathering, including cabinet ministers, army officers and others.

